# Crescentic glomerulonephritis: what’s different in South Asia? A single center observational cohort study

**DOI:** 10.12688/wellcomeopenres.16071.1

**Published:** 2020-07-08

**Authors:** Suceena Alexander, Sabina Yusuf, Gautham Rajan, Elenjickal Elias John, Sanjeet Roy, VC Annamalai, Athul Thomas, Jeethu Joseph Eapen, Anna T Valson, Vinoi George David, Santosh Varughese

**Affiliations:** 1 Department of Nephrology, Christian Medical College, Vellore, Tamil Nadu, 632004, India; 2 Department of General Pathology, Christian Medical College, Vellore, Tamil Nadu, 632004, India

**Keywords:** crescentic glomerulonephritis, rapidly progressive glomerulonephritis, ANCA associated vasculitis, anti-GBM disease, double positive disease, immune complex glomerulonephritis

## Abstract

**Background:** The spectrum and outcomes of crescentic glomerulonephritis (Cr.GN) in South Asia is vastly different from that reported worldwide and there is a paucity of information. The aim of the study was to study the demography, clinical presentation, histology and predictors of longitudinal outcomes of Cr.GN in this population.

**Methods:** An observational cohort study of renal biopsies was performed in the largest tertiary center in South India over a period of 10 years (January 2006 to December 2015) with ≥50% crescents on renal histology indicating Cr.GN.

**Results:** A total of 8645 kidney biopsies were done; 200 (2.31%) were Cr.GN. Patients were categorized into three etiological groups: anti-glomerular basement membrane (type I), immune complex (type II), and pauci-immune (type III). Type II was the most common (96, 46.5%), followed by type III (73, 38%) and type I (31, 15.5%). Female preponderance was seen across all types. About half of all patients presented with recent onset hypertension. Type II had the highest median proteinuria (4.2 (2.1-6) g/day, p=0.06) and the median estimated glomerular filtration rate was lowest in type I (5 (4-8) ml/min/1.73m ^2^, p<0.001). Among type III, anti-neutrophil cytoplasmic antibodies (ANCA)-associated vasculitis was seen only in ~50% of patients. Nearly one third of patients with type I were also positive for ANCA making them ‘double positive’. Acute glomerular insults like tuft necrosis and chronic changes as evidenced by moderate to severe interstitial fibrosis, was a predominant feature of type I.

**Conclusions:** ANCA-negative pauci-immune vasculitis, as well as double positive Cr.GN, are reported for the first time in South-Asia. Renal survival was significantly worse in type I/III compared to type II. Types I/III, moderate to severe interstitial fibrosis and tubular atrophy, presence of oliguria/anuria and increasing percentage of crescents in renal biopsy were significant predictors of end stage kidney disease in our cohort.

## Introduction

Crescentic glomerulonephritis (Cr.GN) is defined histologically by the presence of extensive glomerular crescents (usually greater than 50%). Clinically, it is also known as rapidly progressive glomerulonephritis (RPGN) as it is accompanied by rapid decline in renal functions. It can occur in any glomerular disease ^1^ and is usually reported in about 4% to 10% of native kidney biopsies ^2^. The natural course of the disease is akin to "medical emergency", as end stage kidney disease (ESKD) is reached in most patients within a few weeks to months ^3^. Understanding the clinical presentation, natural history and outcomes of Cr.GN is of major concern for nephrologists worldwide. Various studies have been conducted worldwide and epidemiologic data are available from large national kidney biopsy registries, including those from the United States ^4^, China ^2^, Japan ^5^, Spain ^6^ and Saudi Arabia ^7^. Notably, pathogenesis of glomerular diseases involves a complex and as yet incompletely understood interplay between epigenetic, immunoregulatory, hormonal, and environmental factors on a background of genetic predisposition ^8^. This translates into a broad spectrum of disease presentation, a variable tempo of progression and heterogenous outcomes, which are evident from these previous studies. To this end, exploring the features of Cr.GN in a South Asian population, a genetically and demographically diverse group of individuals, may help to provide useful insights into the causality, and predictors of severe outcomes. Our aim was to study the demography, clinical presentation, histology and predictors of longitudinal outcomes of Cr.GN in this population.

## Methods

### Study design, setting and participants

This was an observational retrospective cohort study performed at the outpatient and inpatient services of the Department of Nephrology, Christian Medical College Vellore, India.

We included all patients (≥18 years) who underwent native renal biopsy at our centre between January 2006 to December 2015 and had ≥50% crescents on renal histology. There were no other inclusion or exclusion criteria.

### Data collection

Data on patients’ demographic profile, clinical features, biochemical parameters, histopathology, treatments, morbidity and mortality were retrieved from the electronic patient records (Clinical WorkStation) maintained in the hospital. Follow-up clinical and outcome data with regards to their serum creatinine, dialysis requirement, and complications were collected for each follow-up visit until August 2016. During the index and follow-up visits, patients were classified into chronic kidney disease (CKD) stages as per estimated glomerular filtration rate (eGFR) calculated by CKD-EPI equation ^9^.

***Data definitions.*** Cr.GN was defined as the presence of ≥50% glomerular crescents as the principal histologic finding. Patients were categorized into three groups on the basis of etiology of Cr.GN; type I, anti- glomerular basement membrane (GBM) Cr.GN; type II, immune complex Cr.GN; type III, pauci-immune Cr.GN.

Microhematuria was defined as >5 red blood cells per high power field. Proteinuria was assessed from 24-hour timed collection as is the standard practice in our center. Interstitial fibrosis and tubular atrophy (IFTA) was classified as follows: focal, <25%; moderate, 25–50%; and severe, >50%.

Qualitative and semiquantitative determination of anti-nuclear antibody (ANA) in serum was done manually by EUROIMMUN Mosaic Hep-20-10 indirect immunofluorescence test (IIFT). The test was done with a sample dilution starting point of 1:100. It is graded on a scale of 1+ to 5+. The sensitivity of the test was 100% with a specificity of 96%. Quantitative determination of anti-double stranded DNA (anti-dsDNA) in serum was done by Anti-dsDNA-NcX ELISA (IgG). The upper limit of the normal range (cut-off) was 100 IU/ml. Anti-neutrophil cytoplasmic antibodies (ANCA) were determined by measuring anti-myeloperoxidase (anti-MPO) and anti-proteinase3 (anti-PR3). Quantitative determination of anti-MPO was done by Anti-Myeloperoxidase ELISA (IgG) test kit. The upper limit of the normal range (cut-off) is 20 RU/ml. The ELISA had a sensitivity of 93.3% and a specificity of 99.8%. Quantitative determination of anti-PR3 was done by Anti-PR3-hn-hr ELISA (IgG). The upper limit of the normal range (cut-off) was 20 RU/ml. The ELISA had a sensitivity of 94% and a specificity of 99%. The tests kits for antibodies were from EUROIMMUN, Luebeck, Germany.

Quantitative determination of complement factors (C3 and C4) was done by means of endpoint nephelometry on the BN ProSpec System by Siemens Health Care Diagnostics Products, Marburg, Germany. Antisera used were liquid animal sera produced by immunization of rabbits with highly purified human complement factors (C3c or C4). The following reference intervals applied for serum samples from healthy adults: C3/C3c, 0.9–1.8 g/L; C4/C4c, 0.1–0.4 g/L.

### Statistical analysis

Data are presented as mean ± standard deviation or medians (interquartile range) or frequency and percent (%) according to the types and distribution of variables. Differences among groups of normally distributed variables were analyzed by t test or one-way analysis of variance (ANOVA). Post-hoc comparisons were performed using t-test with Bonferroni correction. Differences among groups of non-parametric variables were analyzed by Mann–Whitney U-test or the Kruskal-Wallis Test. Categorical variables were compared using chi-squared or Fisher’s exact test. Multivariable logistic regression was used to identify predictors of ESKD.

Statistical calculations were performed using SPSS software for Windows, version 21.0 (SPSS Inc., Chicago, IL) and graphs were made using Graph Pad Prism 7.0e (Graph Pad Software Inc., San Diego, CA). A *P* value of <0.05 was taken as significant.

### Ethical considerations

Approval was obtained from the Institutional Review Board (Silver, Research and Ethics Committee) of the Christian Medical College, Vellore, India (IRB 9090 dated 06.10.2014). Waiver of informed consent was obtained from the ethics committee as the study was retrospective and used de-identified patient information from electronic records.

## Results

### Demography

**Figure 1.**
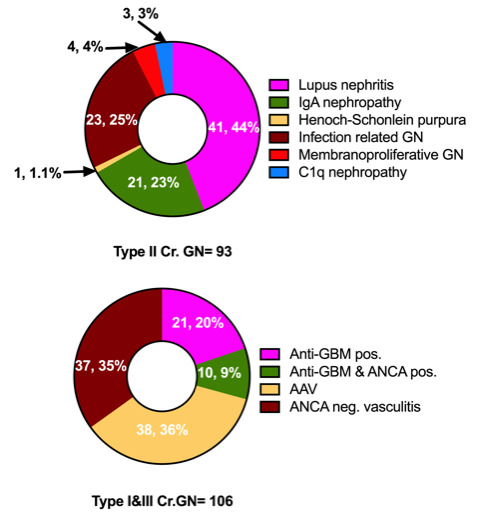
Etiologies of crescentic glomerulonephritis (Cr.GN). GBM, glomerular basement membrane; ANCA, anti-neutrophil cytoplasmic antibodies; AAV, ANCA associated vasculitis.

A total of 8645 kidney biopsies were done at our center from January 2006 to December 2015, of which 200 had Cr.GN (2.31%). The most common cause of Cr.GN was type II (96, 46.5%), followed by type III (73, 38%), and type I (31, 15.5%). The various etiologies of Cr.GN are depicted in Figure 1. Females constituted 60% of the patients with a female: male ratio of 1.5:1. Female preponderance was seen across all three types of Cr.GN. The mean age of presentation for all types was 40.6±14.6 years, with the highest mean age of presentation seen in patients with type III Cr.GN. Demographic and baseline clinical and laboratory parameters of the study population are summarized in Table 1.

**Table 1.** Demography, baseline clinical and laboratory characteristics of the study population.

Characteristic	All types (n=200)	Type I Cr.GN (anti-GBM) (n=31)	Type II Cr.GN (immune complex) (n=93)	Type III Cr.GN (pauci-immune) (n=76)	*P* value
Age (years, mean±SD)	40.6±14.6	37.5±12.2	37.8±14.6	45.3±14.3	**0.03 ^@^, 0.003 ^#^**
Gender (male:female (ratio))	80:120 (0.67)	12:19 (0.63)	36:57 (0.63)	32:44 (0.73)	0.89
Renal symptoms (n (%)) Oliguria Anuria Visible hematuria Non-visible hematuria Hypertension Uremic symptoms	112 (56) 20 (10) 25 (12.5) 190 (95) 100 (50) 84 (42)	23 (74.2) 5 (16.1) 6 (19.4) 29 (93.5) 15 (48.4) 20 (64.5)	50 (53.8) 4 (4.3) 9 (9.7) 90 (96.8) 51 (54.8) 30 (32.3)	39 (51.3) 11 (4.5) 10 (13.2) 71 (93.4) 34 (44.7) 34 (44.7)	0.08 **0.03** 0.38 0.55 0.42 **0.006**
Extra-renal symptoms (n (%)) Skin lesions Arthritis Hemoptysis	24 (12) 40 (20) 19 (9.5)	0 6 (19.4) 4 (12.9)	16 (17.2) 18 (19.4) 0	8 (10.5) 16 (21.1) 15 (19.7)	**0.006** 0.96 **<0.001**
Diabetes mellitus (n, %)	14 (7)	0	5 (5.4)	9 (11.8)	**0.03**
Hemoglobin (g/dL, mean±SD)	8.6±2.1	7.6±1.9	9±2	8.5±2.1	**0.003 ^$^**
Total leucocyte (cells*10 ^9^/L, mean±SD)	9.9±4.5	9.3±3.9	9.8±5.2	10.3±3.7	0.57
Serum cholesterol (mg/dL, mean±SD, n)	199.7±73.8 (123)	205.3±101.8 (15)	213.9±73.2 (62)	178.8±59.6 (46)	**0.04 ^#^**
Serum albumin (g/dL, mean±SD)	3±0.7	3.2±0.6	2.8±0.8	3.1±0.5	**0.008 ^$^, 0.005 ^#^**
24-hour proteinuria (g/day, median (IQR), n)	3.5 (1.8-5.6) (187)	3.3 (2-5.8) (26)	4.2 (2.1-6) (89)	2.8 (1.3-4.3) (72)	**0.06**
Serum creatinine (mg/dL, mean(SD)	6.9±4.4	10.6±5.5	5.1±3.2	7.6±4.1	**<0.001 ^@$^, 0.001 ^#^**
CKD-EPI eGFR (ml/min/1.73m ^2^, median (IQR))	9 (5-16.8)	5 (4-8)	14 (8-25.5)	7 (5-12)	**<0.001 ^@#^**
CKD stages at baseline (n (%)) Stage 2 Yes Stage 3 Yes Stage 4 Yes Stage 5 Yes Stage 5D Yes	7 (3.5) 19 (9.5) 34 (17) 52 (26) 88 (44)	0 1 (3.2) 0 6 (19.4) 24 (77.4)	6 (6.5) 13 (14) 24 (25.8) 21 (22.6) 29 (31.2)	1 (1.3) 5 (6.6) 10 (13.2) 25 (32.9) 35 (46.1)	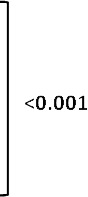
Serum complements (mg/dL, n/N (%)) Low C3 Low C4	84/196 (42.9) 21/196 (10.7)	7/31 (22.6) 0	59/90 (65.6) 20/90 (22.2)	18/75 (24) 1/75 (1.3)	**<0.001** **<0.001**
Serology (n/N (%)) ANA Yes Anti- dsDNA Yes ANCA Yes Anti-MPO-ANCA Yes Anti-PR3-ANCA Yes	51/176 (29) 25/146 (17.1) 54 (31.8) 28/143 (19.6) 26/143 (18.2)	7/31 (22.6) 0 10/31 (32.3) 7/25 (28) 3/25 (12)	38/80 (47.5) 25/75 (33.3) 6/64 (9.4) 3/50 (6) 3/50 (6)	6/65 (9.2) 0 38/76 (50) 18/68 (26.5) 20/68 (29.4)	**<0.001** **<0.001** **<0.001** **<0.001** **<0.001**

*Abbreviations:* Cr.GN, crescentic glomerulonephritis; eGFR, estimated glomerular filtration rate (calculated using the CKD-EPI, Chronic Kidney Disease Epidemiology Collaboration formula); C3, complement C3; C4, complement C4; ANA, anti-nuclear antibody; Anti- dsDNA, anti-double stranded DNA antibody, ANCA, anti-neutrophil cytoplasmic antibody; MPO; myeloperoxidase; PR3, proteinase 3; GBM, glomerular basement membrane.

*P* value is significant at <0.05 between ^@^ Type 1 and Type III, ^#^Type II and Type III, ^$^Type 1 and Type II analyzed by One-way ANOVA with Bonferroni correction.

### Clinical and laboratory features

Non-visible hematuria was near universal (95%). More than half of the patients (56%) were oliguric at presentation. Anuria at presentation was seen in 10% patients. Oliguria and anuria were more common in type I Cr.GN patients (oliguria in 74%, p=0.08; anuria in 16%, p=0.04) who also had significantly more uremic symptoms (64%, p=0.006). About half of all the three types presented with recent onset hypertension. Among extra-renal symptoms, skin lesions and arthritis were rare in type I, but hemoptysis was seen only in type I and III Cr.GN.

Type II Cr.GN had the highest median proteinuria (4.2 (2.1–6) g/day, p=0.06), lowest serum albumin (2.8±0.8 g/dL, p <0.001) and highest serum cholesterol levels (214±73 mg/dL, p=0.04). The median eGFR was 9 (5–17) ml/min/1.73m ^2^ and was lowest in type I Cr.GN (5 (4–8) ml/min/1.73m ^2^, p<0.001) with the lowest mean Hb (7.6±2 g/dL, p=0.003).

Serum complement levels were low in nearly 50% patients with Cr.GN (low C3 in 43% and low C4 in 11%), predominantly in those with type II Cr.GN (p <0.001). Interestingly, low C3 levels were also found in about 25% patients with either type I or type III Cr.GN. Low C4 levels, on the other hand was seen only in type II Cr.GN. Though ANA was positive to various extents in all types of Cr.GN (23% in type I, 9% in type II & 47% in type III), dsDNA positivity was seen only type II Cr.GN (33%). Among type III Cr.GN, ANCA associated vasculitis (AAV) was seen only in 50% patients (anti-MPO 24%; anti-PR3 26.3%). Nearly one third patients with type I Cr.GN (32%) showing linear staining in immunofluorescence were also positive for ANCAs (anti-MPO 22.5%; anti- PR3 9.7%) making them ‘double positive Cr.GN’.

### Histopathological features

Characteristic histopathological features noted in kidney biopsies have been summarized in Table 2. The highest percentage of glomeruli with crescents was seen in type I (83±17%, p=0.001) followed by type III (74±19%) and type II (69±18%). The most common type of crescents in all three groups was fibrocellular. Glomerular proliferative lesions, as evidenced by mesangial hypercellularity (68%, p=0.02), endocapillary proliferation (100%) and neutrophilic exudation (50%, p=0.01), were predominantly seen in type II Cr.GN. Severe glomerular insults, such as tuft necrosis, was a predominant feature of type I (32%, p=0.002) and type III Cr.GN (25%). Chronicity, as evidenced by moderate to severe IFTA, was predominant in type I Cr.GN (68%; p=0.02).

**Table 2.** Histopathology characteristics of the study population.

Characteristic	All types (n=200)	Type I Cr.GN (anti-GBM) (n=31)	Type II Cr.GN (immune complex) (n=93)	Type III Cr.GN (pauci-immune) (n=76)	*P* value
Number of glomeruli (median (IQR))	9 (6-12)	11 (6-15)	9 (6-11)	9.5 (6-12)	0.46
Number of sclerosed glomeruli (median (IQR))	1 (0-3)	0 (0-6)	1 (0-3)	1 (0-4)	0.23
Crescents (%, mean±SD) Predominant type (n (%)) Cellular Cellular to fibro-cellula Fibrocellular Fibrous	73.2±18.9 49 (24.5) 40 (20) 88 (44) 23 (11.5)	83±16.7 5 (16.1) 10 (32.3) 10 (32.3) 6 (19.4)	69.2±18.1 22 (23.7) 18 (19.3) 46 (49.5) 7 (7.5)	74.2±19.4 22 (29) 12 (15.8) 32 (42.1) 10 (13.2)	**0.001 ^$^** 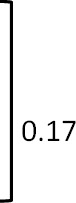
Glomerular lesions (n (%)) Mesangial proliferation Yes Inter-capillary mesangial sclerosis Yes Endocapillary proliferation Yes Neutrophilic infiltration Yes Tuft necrosis Yes Glomerular thrombosis Yes	116 (58) 16 (8) 79 (100) 79 (39.5) 37 (18.5) 3 (1.5)	13 (41.9) 6 (19.3) - 9 (29) 10 (32.2) 0	63 (67.7) 3 (3.2) 79 (100) 47 (50.5) 8 (8.6) 3 (3.2)	40 (52.6) 7 (9.2) - 23 (30.3) 19 (25) 0	**0.02** **0.02** **0.01** **0.002** **0.1**
IFTA Moderate/Severe Yes (n (%))	100 (50)	21 (67.7)	38 (40.9)	41 (53.9)	**0.02**
Vascular (n (%)) Necrosis Yes Arterio(lo)sclerosis Yes	7 (3.5) 55 (23)	1 (3.2) 12 (38.7)	3 (3.2) 27 (29)	3 (3.9) 16 (21.1)	0.96 0.95

*Abbreviations:* IFTA, interstitial fibrosis and tubular atrophy; Cr.GN, crescentic glomerulonephritis; GBM, glomerular basement membrane.

*P* value is significant at <0.05 between ^@^ Type 1 and Type III, ^# ^Type II and Type III, ^$^Type 1 and Type II analyzed by One-way ANOVA with Bonferroni correction.

### Treatment differences

Standard treatment protocols as per KDIGO 2012 ^10^ guidelines were followed for all patients. Nearly half of the patients required dialysis at presentation, with significantly more in type I and type III Cr.GN (81% & 62% respectively, p<0.001). About one fifth of patients received therapeutic plasma exchange (PLEX), and this was significantly more in type I and type III Cr.GN (42% and 26% respectively, p<0.001). The frequency and indications of PLEX are summarized in Table 3. Immunosuppression with/without PLEX was given in 90% of patients with significantly more in type II and III Cr.GN (92% and 91% respectively, p<0.001).

**Table 3.** Treatment characteristics of study population.

Characteristic	All types (n=200)	Type I Cr.GN (anti-GBM) (n=31)	Type II Cr.GN (immune complex) (n=93)	Type III Cr.GN (pauci-immune) (n=76)	*P* value
IS Yes (n (%)) Steroids alone Yes Steroids plus cyclophosphamide Yes Steroids plus other IS Yes PLEX with IS Yes without IS Yes	177 (88.5) 43 (21.5) 100 (50) 33 (16.5) 37 (18.5) 1 (0.5)	22 (71) 2 (35.5) 18 (58.1) 1 (3.2) 12 (38.7) 1 (3.2)	86 (92.5) 29 (31.2) 33 (35.4) 24 (25.8) 5 (5.4) 0	69 (90.8) 12 (15.8) 49 (64.5) 8 (10.5) 20 (26.3) 0	**0.01** **0.003** 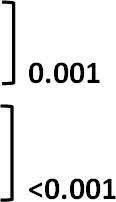
PLEX indications (n (%)) Hemoptysis Yes Renal failure Yes Both Yes	2 (1) 26 (13) 11 (5.5)	1 (3.2) 10 (32) 3 (9.7)	0 5 (5.4) 0	1 (1.3) 11(14.5) 8 (10.5)	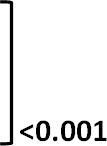
Hemodialysis (n (%))	104 (52)	25 (80.6)	32 (34.4)	47 (61.8)	**< 0.001**

*Abbreviations:* IS, immunosuppression; PLEX, plasma exchange; IS, immunosuppression; CR.GN, crescentic glomerulonephritis; GBM, glomerular basement membrane.

### Patient and renal outcomes

The mean follow-up period was 9.4±15 months. Table 4 summarizes the outcomes observed in three types of Cr.GN. In the entire cohort, nearly half (45%) developed ESKD, requiring renal replacement therapy. This was significantly more in type I (77%, p<0.001). Overall mortality rate was 4% and did not vary significantly between the groups. Sepsis was found to be the most common cause of death.

**Table 4.** Outcomes at follow-up for study population.

Characteristic	All types (n=200)	Type I Cr.GN (anti-GBM) (n=31)	Type II Cr.GN (immune complex) (n=93)	Type III Cr.GN (pauci-immune) (n=76)	*P* value
Follow-up (months, mean±SD)	9.4±15.5	4.6±8.3	10±15.1	10.6±17.8	0.17
Serum creatinine at last follow-up (mg/dL, mean±SD, n)	3.7±3.5 (95)	7.2±5.8 (6)	3.4±3.3 (49)	3.5±3.1 (40)	**0.03 ^@^,** **0.04 ^#^**
eGFR (CKD-EPI) at last follow-up (ml/min/1.73m ^2^, median (IQR), n)	27.5 (11.3-54.5) (95)	11 (4-34) (6)	32 (42.3-33.8) (49)	24.5 (14-43) (40)	0.07
eGFR change from baseline (n/N (%)) eGFR loss & no change in CKD stage eGFR loss & change in CKD stage eGFR gain & no change in CKD stage eGFR gain & change in CKD stage	11/105 (11.6) 10/105 (10.5) 24/105 (25.3) 50/105 (52.6)	3/6 (50) 0 1/6 (16.7) 2/6 (33.3)	3/49 (6.1) 6/49 (12.2) 13/49 (26.5) 27/49 (55.1)	5/40 (12.5) 4/40 (10) 10/40 (13.2) 21/40 (52.5)	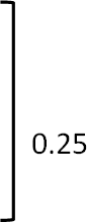
ESKD	89 (44.5)	24 (77.4)	28 (30.1)	38 (50)	**<0.001**
Hemodialysis (n (%))	87 (43.5)	24 (77.4)	28 (30.1)	35 (46)	**<0.001**
Infections (n (%))	34 (17)	5 (16.1)	19 (20.4)	10 (13.2)	0.45
Death	9 (4.5)	0	4 (4.3)	5 (6.6)	0.17

*Abbreviations:* eGFR, estimated glomerular filtration rate (calculated using the CKD-EPI, Chronic Kidney Disease Epidemiology Collaboration formula); ESKD, end stage kidney disease; Cr.GN, crescentic glomerulonephritis; GBM, glomerular basement membrane.

*P* value is significant at <0.05 between ^@^ Type 1 and Type III, ^#^Type II and Type III, ^$^Type 1 and Type II analyzed by One-way ANOVA with Bonferroni correction.

### Type III Cr.GN: ANCA associated vasculitis (AAV) vs. ANCA negative vasculitis

**Figure 2.**
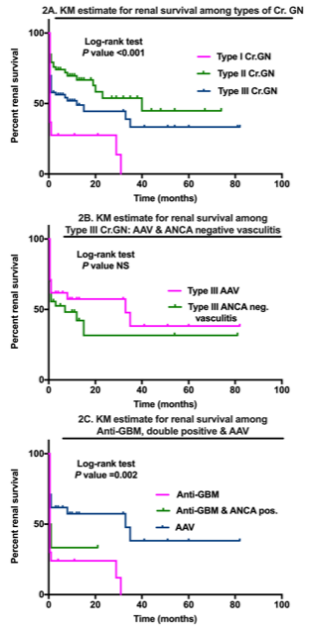
Kaplan-Meier estimates for renal survival in types of crescentic glomerulonephritis and their subgroups.

The group of patients with type III Cr.GN was subdivided into AAV (38, 51%) and ANCA negative (37, 49%) vasculitis. Characteristic features of the two groups are summarized in Table 5. AAV was associated with a significantly higher mean age (p=0.01), presence of extra renal manifestations of fever (50%, p=0.02), hemoptysis (39%, p<0.001) leukocytosis (11.2±4.5 cells*10 ^9^/L, p=0.03), severe histology, as evidenced by fibrous/ fibrocellular crescents (84%, p=0.009), tuft necrosis (37%, p=0.02), and greater requirement of PLEX (45%, p<0.001). However, the median 24-hour proteinuria (3.7 (2.1–6.7) g/day, p=0.001) and serum cholesterol (206.7±61 mg/dL, p=0.003) was higher in ANCA negative vasculitis. Rates of ESKD and renal survival did not differ between the two groups ( Table 5 and Figure 2).

**Table 5.** Type III Cr.GN: characteristics of AAV and ANCA negative vasculitis.

Characteristic	ANCA neg. vasculitis(n=37)	AAV(n=38)	*P* value
Age (years, mean(SD)	41±13.3	49.4±14.4	**0.01**
Gender (male:female (ratio))	15:22 (0.68)	16:22 (0.73)	0.89
Renal symptoms (n (%)) Oliguria Anuria Hypertension Uremic symptoms	20 (54.1) 6 (16.2) 19 (51.4) 18 (48.6)	18 (47.4) 4 (10.5) 14 (36.8) 15 (39.5)	0.56 0.52 0.21 0.42
Extra-renal symptoms (n (%)) Skin lesions Arthritis Hemoptysis Fever	3 (8.1) 5 (13.5) 0 9 (24.3)	5 (13.2) 11 (28.9) 15 (39.5) 19 (50)	0.71 0.1 **<0.001** **0.02**
Hemoglobin (g/dL, mean±SD)	8.8±2.7	8.2±2	0.19
Total leucocyte (cells*10 ^9^/L, mean±SD)	9.3±2.5	11.2±4.5	**0.03**
Serum cholesterol (mg/dL, mean±SD, n)	206.7±61.1	155.3±47.9	**0.003**
Serum Albumin (mg/dL, mean±SD)	3.1±0.5	3.1±0.6	0.79
24-hour proteinuria (g/day, median (IQR), n)	3.7 (2.1-6.7)	2.1 (0.9-3.5)	**0.001**
Serum creatinine (mg/dL, mean±SD)	7.1±4	7.9±4.3	0.39
CKD-EPI eGFR (ml/min/1.73m ^2^, median (IQR))	9 (5.5-16)	7 (4-10.3)	0.11
Serum complements (mg/dL, n/N (%)) Low C3 Low C4	12 (32.4) 1 (2.7)	6 (16.2) 0	0.1 1
Crescents (%, mean±SD) Predominant type (n (%)) Cellular Fibrocellular or fibrous	74.8±20.5 16 (43.2) 21 (56.8)	73±18.3 6 (15.8) 32 (84.2)	0.7 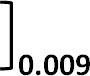
Glomerular lesions (n (%)) Mesangial proliferation Yes Neutrophilic infiltration Yes Tuft necrosis Yes	20 (54.1) 12 (32.4) 5 (13.5)	20 (52.6) 11 (28.9) 14 (36.8)	0.9 0.74 **0.02**
IFTA Moderate/Severe Yes (n, (%))	20 (54.1)	20 (52.6)	0.9
Vascular (n (%)) Necrosis Yes Arterio(lo)sclerosis Yes	1 (2.7) 26 (70.3)	2 (5.3) 21 (55.3)	0.57 0.18
Treatment (n (%)) Immunosuppression Yes PLEX Yes	33 (89.2) 3 (8.1)	36 (94.7) 17 (44.7)	0.38 **<0.001**
Outcomes Serum creatinine at last follow-up (mg/dL, mean±SD, n) eGFR at last follow-up (ml/min/1.73m ^2^, median (IQR), n) ESKD Death	4±4.2, 18 29 (17.8-51.7), 18 20 (54.1) 0	3.1±1.9, 22 21.6 (13.2-34.7), 22 17 (44.7) 5 (13.2)	0.42 0.09 0.42 0.07

*Abbreviations:* ANA, anti-nuclear antibody; ANCA, anti-neutrophil cytoplasmic antibody; eGFR, estimated glomerular filtration rate (calculated using the CKD-EPI, Chronic Kidney Disease Epidemiology Collaboration formula); ESKD, end stage kidney disease; C3, complement C3; C4, complement C4; IFTA, interstitial fibrosis and tubular atrophy; PLEX, plasma exchange; ESKD, end stage kidney disease; Cr.GN, crescentic glomerulonephritis; GBM, glomerular basement membrane.

### Double antibody positive Cr.GN

Among those with type I Cr.GN (n=31), there were 10 ‘double positive’ patients (32.3%) with positive ANCA serologies (anti-MPO 7; anti-PR3 3). Renal survival was significantly worse in this group (p=0.002) compared to AAV but similar to type I Cr.GN ( Figure 2).

### Predictors of severe outcomes

We analyzed various risk factors which predicted the development of ESKD at follow-up in this cohort ( Table 6). In an adjusted regression analysis, type I/type III Cr.GN, moderate to severe IFTA, presence of oliguria/anuria and increasing percentage of crescents in renal biopsy were significant predictors of ESKD at follow-up.

**Table 6.** Significant predictors of ESKD at follow-up.

Risk factors	Univariate *P* value	Multivariable regression			
		Exp (B)	95% C.I.		*P* value
			Lower	Upper	
Gender	0.22				
Renal symptoms Oliguria Yes Anuria Yes Uremic symptoms Yes	**<0.001** **0.004** **<0.001**	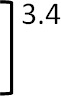	1.7	6.7	**<0.001**
Extra-renal symptoms Skin lesions No Arthritis No	0.038 0.014				
Serum creatinine (mg/dL)	**<0.001**				
CKD-EPI eGFR (ml/min/1.73m2)	**<0.001**				
Normal C3	0.66				
Normal C4	**0.04**				
Serology ANA neg. Anti- dsDNA pos. ANCA pos.	**0.014** 0.07 0.68				
Percentage of crescents	<0.001	1.02	1.005	1.04	**0.01**
Glomerular lesions Mesangial proliferation No Predominant type of crescents Cellular Yes Vascular Necrosis Yes Interstitial IFTA Moderate/Severe Yes	**0.01** 0.28 **0.022** **<0.001**	3.4	1.8	6.5	**<0.001**
Type I/III Cr.GN	**<0.001**	2.7	1.4	5.1	**0.003**
Immunosuppression No	**0.003**				
PLEX Yes	0.1				
Hemodialysis at index visit discharge Yes	**<0.001**				

*Abbreviations:* ANA, anti-nuclear antibody; ANCA, anti-neutrophil cytoplasmic antibody; C3, complement C3; C4, complement C4; IFTA, interstitial fibrosis and tubular atrophy; PLEX, plasma exchange; CR.GN, crescentic glomerulonephritis; eGFR, estimated glomerular filtration rate (calculated using the CKD-EPI, Chronic Kidney Disease Epidemiology Collaboration formula).

**Figure 3.**
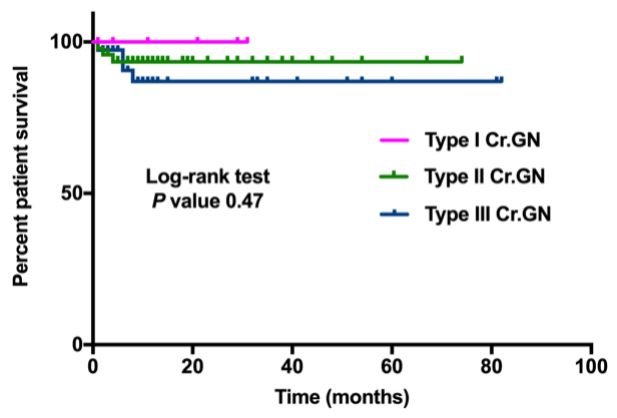
Kaplan-Meier estimate for patient survival in types of crescentic glomerulonephritis.

Kaplan-Meier estimates of renal survival differed significantly between the three groups ( Figure 2) but did not differ in any subgroups ( Figure 2). Patient survival was similar in all three groups ( Figure 3).

## Discussion

Cr.GN is one of the leading causes of rapidly progressive renal failure. There are a few studies of Cr.GN from South Asia but there is dearth of data on the different types of Cr.GN and their outcomes.

In this study Cr.GN accounted for 2.3% of all biopsies conducted over a period of 10 years, which is comparable to previously reported rates of Cr.GN from India ^11, 12^. Type II Cr.GN was found to be the most common type, which is unique to the Asian continent. Various studies conducted in different parts of the world have shown type III Cr.GN to be the most common ^5, 13^. However studies from China, Saudi Arabia and a smaller Indian study have reported type II Cr.GN to be the most common ^2, 11, 14^. Reasons cited for increased incidence of type II Cr.GN in these parts have been an increased incidence of infections and a higher prevalence of IgA nephropathy. Lupus nephritis (45%) is the most common cause of type II Cr.GN, followed by infection related GN (24%) and IgA nephropathy (23%) in our cohort. This is similar to one of the largest reviews of Cr.GN from China, in which lupus nephritis was listed as most common cause of type II Cr.GN (34%) followed by IgA nephropathy (17%) ^2^.

Gender differences in Cr.GN have mostly been observed in type II Cr.GN with female preponderance. The gender distribution has been variable in type I and type III Cr.GN in different studies ^2, 11, 13– 15^. As is already known and established in other studies, patients with type I Cr.GN had the most severe renal failure at presentation. However, in this study, more than half of the patients with type II and III Cr.GN also presented with severe renal failure, highlighting the dramatic presentation in this patient population. These rates are much higher than previously reported ^2, 15^.

Serum complement C3 levels have been shown to be low in type I and type III Cr.GN in addition to type II Cr.GN. This was confirmed in our study. However, we found no cases of low C4 levels in type I and III Cr.GN, highlighting that the alternate complement pathway has a role in the pathogenesis of these GNs. Our observed rates of ANCA seropositivity in type III Cr.GN are much lower than those reported in other studies ^2, 15^. An earlier study from India also reported similar rates of ANCA seropositivity ^16^. ANCA negative vasculitis could be attributed to other antibodies, such as anti-endothelial cell antibodies, or to cell mediated immune mechanisms, which lead to neutrophilic activation ^17^. The high prevalence of ANCA negative vasculitis in this cohort highlights the need for research into its pathogenesis to elucidate factors specific to our population.

Subgroup analysis of type III Cr.GN revealed important differences between AAV and ANCA negative vasculitis groups, which, to the best of our knowledge, is reported for the first time from India. Similar to our study, younger age of onset and lower prevalence of systemic involvement in ANCA negative vasculitis has been reported from other parts of the world ^18– 21^. Chen *et al*. ^19^ also observed a higher level of proteinuria in this group, similar to our study. Although chronic lesions on kidney biopsy were more prevalent in ANCA negative vasculitis in these studies, we found a higher prevalence of fibrous/fibrocellular crescents and tuft necrosis in the AAV group. Data on renal outcomes has been variable, with few studies showing comparable outcomes in the two groups ^20, 21^, which is similar to our study and others showing poorer renal outcome in ANCA negative vasculitis ^18, 19^.

Levy *et al*. ^22^ reported a prevalence of ANCA positivity of nearly 30% in type I Cr.GN with predominance of anti-MPO ANCA. They concluded that these patients had a poor prognosis when presenting with severe disease and initially behaved more like anti-GBM disease than vasculitis with low rates of recovery from renal failure. On the other hand McAdoo *et al*. ^23^ in their retrospective analysis of double antibody positive cases found that such patients had shared characteristics of AAV and anti-GBM disease. Double positive patients had a greater likelihood of independence from dialysis despite more chronicity compared to patients with anti-GBM disease and long term renal survival was intermediate compared to the single-positive patients. Our prevalence of ‘double positive Cr.GN’ was similar to Levi *et al*. ^22^ with intermediate risk for renal survival.

At the end of the follow-up period, almost half of the patients had developed ESKD in our study. Renal survival was worst in type I Cr.GN. Similar rates of renal survival were reported by Chen *et al*. ^2^ Previously reported rates of renal failure from India varied between 46–60% ^11, 16^. Different studies have identified various risk factors for ESKD: sclerosed glomeruli, acute tubular necrosis, vasculopathy ^7^, arteriolar fibrinoid necrosis ^16^, serum creatinine, age, lung involvement, serum c-reactive protein ^5^, oliguria, crescents and interstitial inflammation ^2^.

## Conclusion

We were able to analyse detailed demographic, clinical, serological and pathological features of our cohort. Strengths of our study include its large sample size, the inclusion and comparison of ANCA negative vasculitis as well as ‘double positive Cr.GN’ patients, of which there is no previously reported data from India. We highlight several important clinical practice points, in particular type II Cr.GN may present with a severe renal failure similar to type III Cr.GN. However, the response to treatment and outcomes were much more favourable and appropriate treatment should be initiated at the earliest. Prevalence of ANCA negative vasculitis was much higher in our population and hence kidney biopsy is mandatory in a case of suspected RPGN with negative serologies. Type I/type III Cr.GN, moderate to severe IFTA, presence of oliguria/anuria, and increasing percentage of crescents in renal biopsy were significant predictors of ESKD at follow-up in our cohort. Our study has also been able to identify areas of further research: search for pathways of alternate complement activation in type I and III Cr.GN and to explore the pathogenesis and factors responsible for increased prevalence of ANCA negative vasculitis in our population. The limitations of the study are that it is a single centre and it was retrospective in nature.

## Data availability

### Underlying data

Figshare: Crescentic GN_repository.xlsx, https://doi.org/10.6084/m9.figshare.12479402.v4 ^24^.

Data are available under the terms of the Creative Commons Attribution 4.0 International license (CC-BY 4.0).
