# Supplementary material for: Crescentic glomerulonephritis: what’s different in South Asia? A single center observational cohort study
Source: Wellcome Open Res. 2020 Jul 8;5:164. [Version 1] doi: 10.12688/wellcomeopenres.16071.1 (PMC7385543; doi:10.12688/wellcomeopenres.16071.1)
Supplement: Peer review history

# Peer review: Reviewer response for version 1

**DOI:** 10.21956/wellcomeopenres.17633.r39470

**Published:** 2020-07-27

**Authors:** Stephen Holdsworth

**Affiliations:** 1 Immunology Department, Monash Health, Clayton, Australia

This is a well performed study of rapidly progressive Crescentic Glomerulonephritis (Cr.GN) from a single hospital in South India. The study surveyed 8645 renal biopsies from 2006-2015 and studied 200 patients who had a biopsy showing Cr.GN (>50% crescentic glomeruli).

 The frequency of this disease amongst biopsied patients with glomerulonephritis (2.3%) is a lower frequency than has been found in other large studies. However this was a relatively large group (200) enabling more confidence in the data collected. 

 Relatively to other large studies there were several significant observations. The relative frequency of infection as an underlying disease was unusually high. There was also a relatively higher than expected frequency of Lupus Nephritis. 

 The other unexpected findings were the high percentage of ANCA negative patients amongst the Type III pauci immune group. These two groups, (Type III ANCA positive and negative), were similar in most clinical and biochemical features except for the observation that ANCA negative patients were significantly younger, had significantly less extra renal disease especially haemoptysis. Consequently they had significantly less plasma exchange treatment. Their biopsies showed less glomerulosclerosis and full glomerular tuft necrosis and they had a strong trends to outcomes with less loss of renal function and death. 

 The frequency of Type I, anti GBM disease group was, as expected, infrequent. Only 21 patients had “pure” anti-GBM disease but the number of double positive (10 patients with Type I anti GBM disease and concurrently Type III classification) significantly raised the percentage of patients with anti GBM disease. If we deleted these 10 double positive patients from the Type III group this “Pure Type III” group would now fall to 68 and the number of ANCA positive patients in this “Pure Type III group” would fall to 28. Therefore the percentage of “pure Type III “patients ANCA positive would fall to 41%. This is a remarkably low frequency of ANCA positivity in Type III Cr.GN patients. It would be interesting to see how many of these patients (“Pure” anti GBM disease, “Pure Type III and” double positive) had circulating anti-GBM antibodies.

 Regarding treatment and outcomes there was an unexpected use of prednisolone alone amongst a group of patients who may have benefitted from immunosuppression with drugs including Rituximab, Mycophenolate and azathioprine. Having made this observation it is important to say that the overall outcomes seem to be similar to those reported in other large patient surveys.

 This is an important study that has demonstrated several novel differences in this group of Cr.GN patients not seen in other large studies. A validation study, also from Southern India, has the potential to strengthen these findings.

Is the work clearly and accurately presented and does it cite the current literature?

Yes

If applicable, is the statistical analysis and its interpretation appropriate?

Yes

Are all the source data underlying the results available to ensure full reproducibility?

No source data required

Is the study design appropriate and is the work technically sound?

Yes

Are the conclusions drawn adequately supported by the results?

Yes

Are sufficient details of methods and analysis provided to allow replication by others?

Yes

Reviewer Expertise:

Immunology.

I confirm that I have read this submission and believe that I have an appropriate level of expertise to confirm that it is of an acceptable scientific standard.

# Response

**Published:** 2020-07-27

**Authors:** Suceena Alexander

**Affiliations:** Christian Medical College, Vellore, India

Dear Dr Stephen

Thank you for your review and comments. I have addressed the reasons for steroid alone treatment in some patients with Cr.GN in my response to Dr. Salama's comments. 

Regards

Suceena Alexander

# Peer review: Reviewer response for version 1

**DOI:** 10.21956/wellcomeopenres.17633.r39463

**Published:** 2020-07-21

**Authors:** Alan Salama

**Affiliations:** 1 Department of Renal Medicine, University College London, London, NW3 2PF, UK

This is an interesting description of a large case series of patients with cresentic nephritis from Southern India. The predominance of immune complex disease is as previously reported and in keeping with higher levels of infection related disease, SLE and IgA. The most interesting features are the high rates of ANCA negative pauci immune GN. The cause of this is uncertain and warrants more investigation, especially as many patients were also hypocomplementaemic - begging the question if these are more like pauci immune lupus like disease or if the immunoglobulin staining was verified by repeat staining. However, the nature of the antigen (and antibody if one exists) would be of great interest. These are experiments beyond the scope of this work, but are intriguing.

 There were many patients treated with steroids alone across all groups (up to a third of group 1 and 2) which is not recommended in KDIGO and would be worth investigating the reasons for, as that may account for some of the ESRD.

Is the work clearly and accurately presented and does it cite the current literature?

Yes

If applicable, is the statistical analysis and its interpretation appropriate?

Yes

Are all the source data underlying the results available to ensure full reproducibility?

No source data required

Is the study design appropriate and is the work technically sound?

Yes

Are the conclusions drawn adequately supported by the results?

Yes

Are sufficient details of methods and analysis provided to allow replication by others?

Yes

Reviewer Expertise:

Immune mediated kidney disease, autoimmunity, glomerulonephritis.

I confirm that I have read this submission and believe that I have an appropriate level of expertise to confirm that it is of an acceptable scientific standard.

# Response

**Published:** 2020-07-23

**Authors:** Suceena Alexander

**Affiliations:** Christian Medical College, Vellore, India

Dear Dr Alan Salama

Thank you for your review and comments. With regards to the steroid alone treatment in group I (anti-GBM), the treatment table shows 2/31 which is 6.5%. The percentage shown in the bracket is a typo and we apologise for the error. In Group II (immune complex), the major reasons for steroid alone treatment were chronicity and severe IFTA on biopsy and the presence of concomitant infections as most were infection related glomerulonephritis. 

Regards

Suceena Alexander
